# Acute Chylous Ascites Status Post Median Arcuate Ligament Syndrome Decompression: A Unique Case Report and Literature Review

**DOI:** 10.7759/cureus.35300

**Published:** 2023-02-22

**Authors:** Leo Sakai, Paul Aguilera, Sathish Karmegam

**Affiliations:** 1 Internal Medicine, Medical City Denton, Denton, USA

**Keywords:** peritoneal fluid analysis, case report, median arcuate ligament syndrome, paracentesis, chylous ascites

## Abstract

Chylous ascites (CA) are a rare finding of triglyceride-rich peritoneal fluid within the abdominal cavity. Malignancy, cirrhosis, and trauma after abdominal surgery are the leading causes of CA in adults. Currently, there are no published guidelines on the management of CA. This report describes a case of an 18-year-old female presenting with abdominal pain and distention following median arcuate ligament syndrome (MALS) decompression. A computed tomography (CT) of the abdomen and pelvis showed large-volume ascites with normal hepatic morphology. Paracentesis and ascitic fluid studies were positive for milky fluid rich in triglyceride. Her recent history of MALS decompression revealed the cause of her acute CA to be a postoperative complication from her abdominal surgery. This case highlights the diverse etiology of ascites and the importance of a careful history and physical examination when evaluating adults with ascites.

## Introduction

Chylous ascites (CA) are a collection of triglyceride-rich fluid in the abdominal cavity, characterized by a milky appearance. It develops as a result of disruption to the lymphatic system due to direct traumatic injury or obstruction. Herein, we report a case of acute CA suspected to be caused by median arcuate ligament syndrome (MALS) decompression surgery.

## Case presentation

An 18-year-old female with a past surgical and medical history of asthma and MALS for which she underwent robotically assisted median arcuate ligament decompression presented with complaints of abdominal pain and distention. Her symptoms started two weeks prior to admission and the patient has never experienced these symptoms before. Her abdominal pain and distention gradually worsened over the last couple of weeks, which ultimately prompted her to visit the emergency department (ED). She recalled no inciting event, apart from having her MALS surgery performed three weeks ago. Nothing aggravates or alleviates her symptoms. The patient described her symptoms as generalized abdominal pain and denied any signs of radiation. The family history was unremarkable. The patient denied tobacco, alcohol, or illicit drug use. Her travel history is only significant for a recent trip to California. Her home medications include budesonide/formoterol (Symbicort) and albuterol (ProAir) inhaler.

Upon presentation, the patient was found to be afebrile with a temperature of 98.0°F (36.6°C), tachycardic with a heart rate of 126 beats per minute, hypertensive with a blood pressure of 151/88 mmHg, respiratory rate of 20 breaths per minute, and oxygen saturation of 98% on room air. On physical exam, the patient’s abdomen was grossly distended and diffusely tender on palpation with no rebound tenderness or abdominal masses. Bilateral lower extremity edema was also present. Her complete blood count revealed thrombocytosis with a platelet count of 726,000 cells/µL with no signs of anemia or leukocytosis. Her complete metabolic panel showed hypokalemia with potassium of 3.4 mEq/L and hyperglycemia with glucose of 117 mg/dL (Table 1). The liver panel was unremarkable. Additional testing included a negative serum hCG.

**Table 1:** Laboratory Results µL: microliter; g: gram; dL: deciliter; mEq: milliequivalent; mg: milligram; L: liter; U: units

Analyte	Results	Reference Range
Complete Blood Count:		
White blood cell count	10,800 cells/µL	4,8000-10,800 cells/µL
Hemoglobin	13.6 g/dL	12.5-16.0 g/dL
Hematocrit	38.4%	37.0-50.0%
Platelet count	726,000 cells/µL	150,000-450,000 cells/µL
Complete Metabolic Panel:		
Sodium	138 mEq/L	136-145 mEq/L
Potassium	3.4 mEq/L	3.5-5.1 mEq/L
Chloride	104mEq/L	98-107 mEq/L
Bicarbonate	28 mEq/L	22-29 mEq/L
Blood urea nitrogen	15 mg/dL	7-18 mg/dL
Creatinine	1.01 mg/dL	0.6-1.0 mg/dL
Glucose	117 mg/dL	70-110 mg/dL
Alkaline phosphatase	61 U/L	47-119 U/L
AST	27 U/L	10-35 U/L
ALT	38 U/L	12-78 U/L
Total bilirubin	0.2 mg/dL	0.2-1.0 mg/dL

**Figure 1:**
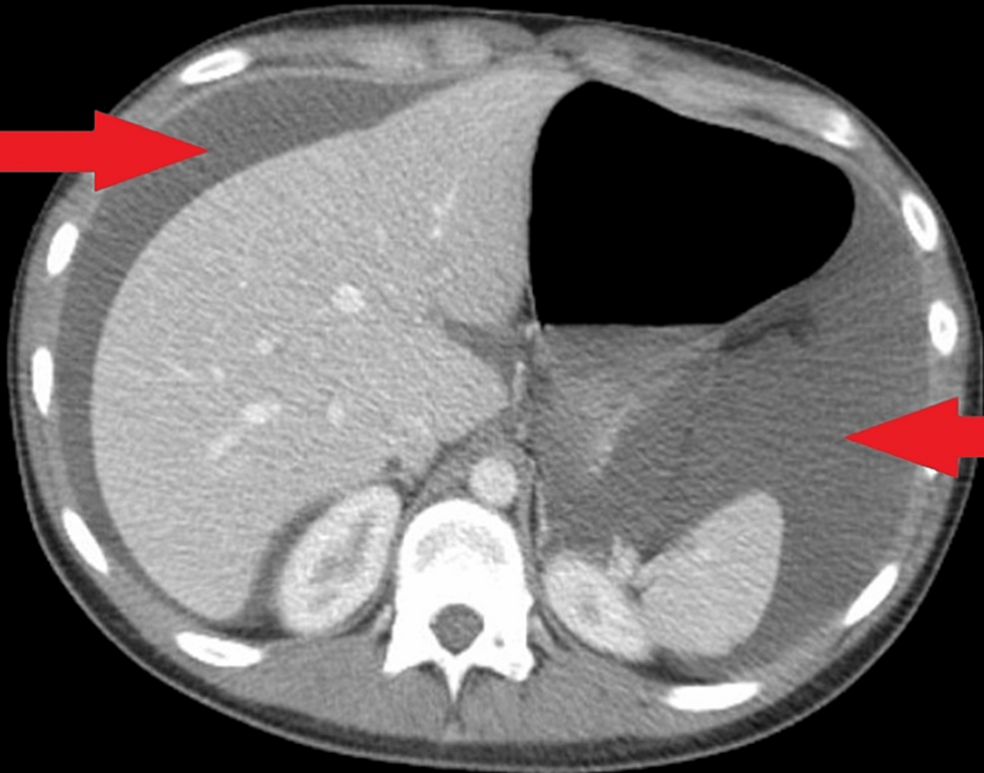
Axial CT of the abdomen/pelvis shows ascites (red arrows).

Initial chest x-ray revealed no acute cardiopulmonary abnormalities. Patient underwent CT imaging of the abdomen/pelvis (Figure 1), which was positive for large volume ascites with normal hepatic morphology. Abdominal paracentesis removed approximately 50ml of cloudy fluid, which was sent for fluid studies (Table 2).

**Table 2:** Paracentesis fluid study g: gram; dL: deciliter; mEq: milliequivalent; mg: milligram; L: liter; U: units

Test	Results
Fluid Color	Creamy
FLD WBC	4878/mm3
FLD RBC	119/mm3
FLD POLY	22%
FLD LYMPH	67%
FLD MONO	11%
Peritoneal FLD Triglyceride	654.0 mg/dL
Peritoneal FLD Total Protein	4.0 g/dL (0.94-6.56 g/dL)
Peritoneal FLD Amylase	20 U/L (6-123 U/L)
Peritoneal FLD Total Bilirubin	<0.2 mg/dL
Peritoneal FLD Culture	No growth after 72 hrs

The patient was started on pain management (tylenol and morphine), antiemetics (ondansetron, prochlorperazine, and diphenhydramine), and diuretic therapy (furosemide). Interventional radiology was also consulted for further image-guided fluid drainage and removed 2.5L of chylous fluid. Patient’s hospital stay was uneventful. She was administered intravenous antibiotic (ceftriaxone) along with subcutaneous octreotide. Lymphatic duct injury was considered given her recent history of abdominal surgery. Patient responded well to treatment with significant improvement in her symptoms. She was discharged after two days with an appointment scheduled to follow up with her operating surgeon as an outpatient.

Prior to her follow-up appointment, however, patient noticed a reoccurrence of her abdominal distention and returned to the ED. An additional 3.8L of fluid was drained and patient was started on a high-protein and low-fat diet.

## Discussion

CA is a rare condition of triglyceride-rich peritoneal fluid within the abdominal cavity [1]. One large university-based hospital measured the cumulative incidence of approximately 1 per 20,000 admissions [2]. However, the exact prevalence of CA today is unknown because no further epidemiological studies have been recently conducted. Many investigators believe the incidence of CA is higher than previously reported due to more aggressive thoracic and abdominal surgeries being performed and cancer patients living longer with advancements in medicine [3]. There are numerous causes of CA, but malignancy, cirrhosis, and trauma after abdominal surgery have been identified as the leading causes of CA in adults [4]. In one systematic review, the most common presenting symptom of CA was abdominal distention, which is consistent with our case. Other clinical manifestation includes nonspecific abdominal pain, diarrhea, dysphagia, peripheral edema, nausea, vomiting, early satiety, and fever [5]. CA can lead to significant morbidity and mortality because a continuous leak in the chyle, which is rich in nutrients and immunoglobulins, can lead to dehydration, malnutrition, electrolyte imbalance, and immunosuppression [4]. The breadth of etiologic possibilities, nonspecific presentation, and significant complications associated with CA highlights the importance of having a stepwise approach to its diagnosis and management.

First-line diagnostic workup for any patient presenting with ascites should include a careful history and physical examination. Detailed history regarding family history, surgical history, travel history, social history, and past medical history, specifically pertaining to malignancy, liver, or renal disease should be obtained [4]. On physical exam, the presence of flank dullness is the most helpful finding and can be typically found in any patient with ascites regardless of underlying etiology [6]. Patients with specific causes of ascites may have stigmata of cirrhosis, malignancy, or heart failure on physical exam. The next step in evaluating patients suspected of having ascites is abdominal imaging with either an ultrasound or CT. Although abdominal CT has been considered the modality of choice to identify the presence of ascites, ultrasound has been shown to be a reliable cost-effective imaging modality as well. One advantage of ultrasound in the detection of ascites is that it can qualitatively distinguish between a simple clear fluid, which appears homogeneously anechoic, and a complex chylous fluid, which demonstrates particulate material or appears layered. In contrast, on CT, both simple and complex fluids will have a uniform hypodense appearance, and one cannot be able to differentiate between chylous and clear ascites [7]. Following the diagnosis of ascites, abdominal paracentesis is the most important diagnostic tool in evaluating the cause. Initial ascitic fluid testing should include an appearance assessment, cell count and differential, and total protein concentration. A serum-to-ascites albumin gradient (SAAG) can also be calculated to help identify whether portal hypertension is the underlying cause of ascites. Triglyceride levels in the ascitic fluid are critical in defining CA with a typical cut-off value of above 200mg/dL, which our patient met (654.0 mg/dL) [1]. Lymphangiography is considered the gold standard in defining cases of obstruction. However, this procedure's utility may be limited by complications such as tissue necrosis, fat embolism, and hypersensitivity to contrast [8].

The management of CA is a multifaceted and individualized process; the mainstay of treatment is to address the underlying cause [9]. Specific treatments have not been well studied. Surgery may benefit the patient with postoperative causes. However, a systematic review showed that conservative management was effective in the majority of cases of postoperative CA [10,11]. A reasonable initial approach in patients with an unidentifiable cause or those not responding to the treatment is to start a high-protein and low-fat diet with medium-chain triglycerides (MCT). The idea behind this dietary restriction is that median-chain triglycerides bypass the lymphatic system and are absorbed by the enterocytes and transported directly to the liver via the portal vein thus reducing the production of chyle [12]. Several case reports have also demonstrated efficacy of using somatostatin and octreotide to successfully treat CA. The exact mechanism is not completely understood, but it is thought that these agents act by decreasing intestinal absorption of fat via specific receptors [13-15]. Another case report showed successful treatment of CA using orlistat in patients unable to comply with dietary restrictions. Orlistat works by inhibiting gastric and pancreatic lipase leading to decreased fat absorption [16]. In patients with large volume ascites, paracentesis should be performed and repeated as needed for symptomatic relief [9]. Overall, a step-up approach should be taken starting with conservative management and medical therapy before moving to more invasive steps.

## Conclusions

This case report highlights the clinical, radiographic, and laboratory features of acute CA, a rare finding due to the disruption of the lymphatic system. This case also highlights the importance of obtaining a careful history when evaluating patients suspected of having ascites. Paracentesis is the gold standard diagnostic tool for the diagnosis of CA with an ascitic fluid triglyceride level above 200mg/dL being the cut-off value to define CA. What makes the management of CA challenging is that there are currently no universally adopted guidelines and therapeutic options are limited, emphasizing the need for treatment to be individualized. The cornerstone of treatment for CA is to address the underlying cause. A reasonable initial approach is to recommend a high-protein and low-fat diet with medium-chain triglycerides. Octreotide, somatostatin, and orlistat have been demonstrated to be efficacious in the treatment of CA. Further controlled clinical trials on these specific agents compared to dietary restriction alone are warranted and investigation of CA refractory to conservative treatment could yield critical information.
